# Conversion of failed proximal femoral nail antirotation to uncemented or cemented femoral component fixation: a multicentre retrospective study with a median 10-year follow-up

**DOI:** 10.1186/s12891-022-05323-8

**Published:** 2022-04-21

**Authors:** Wenbo Shi, Yaodong Zhang, Yangkai Xu, Xianshang Zeng, Hongjing Fu, Weiguang Yu

**Affiliations:** 1 Department of Anesthesiology, Wuhan Fourth Hospital; Puai Hospital, Tongji Medical College, Huazhong University of Science and Technology, No.473, Hanzheng Street, Qiaokou District, Wuhan, 430033 China; 2 Department of Anesthesiology, The First Affiliated Hospital, Sun Yat-Sen University, No. 58, Zhongshan 2nd Road, Yuexiu District, Guangzhou, 510080 China; 3 Department of Orthopedics, Fuzhou Second Hospital Affiliated to Xiamen University, Cangshan District, Fuzhou, China; 4 Department of Orthopedics, The First Affiliated Hospital, Sun Yat-Sen University, No. 58, Zhongshan 2nd Road, Yuexiu District, Guangzhou, 510080 China

**Keywords:** Conversion, Total hip arthroplasty, Revision, Outcome, Complication

## Abstract

**Background:**

Conversion of a failed proximal femoral nail antirotation (PFNA) to a total hip arthroplasty (THA) is becoming increasingly universal. However, consensus has not been reached regarding which device (uncemented or hybrid THA) to use. The aim of this retrospective study was to compare the clinical outcomes of the conversion of failed PFNAs to uncemented versus hybrid THAs in the elderly population.

**Methods:**

Consecutive elderly patients with prior failed PFNAs treated with uncemented or hybrid THA from January 2008 to December 2019 were retrospectively identified from two medical centres. The primary outcome was implant survival after THA revision; secondary outcomes were the functional outcomes assessed using the Harris Hip Score (HHS) and the incidence of key THA-related complications.

**Results:**

A total of 236 patients (uncemented THA, *n* = 116; hybrid THA, *n* = 120) were eligible for this study. Kaplan–Meier survival curves demonstrated that the 10-year cumulative survival rates were 0.801 (95% confidence interval [CI], 0.783–0.852) in the uncemented THA group versus 0.925 (95% CI, 0.861–0.964) in the hybrid THA group (hazard ratio [HR] 0.36 [95% CI 0.24–0.56], *p* = 0.004). From the 72nd month after the revision to the last follow-up, functional outcomes differed considerably between cohorts (each *p* < 0.05), and the rate of key THA-related complications was comparable between cohorts (*p* = 0.004).

**Conclusion:**

For elderly patients with prior failed PFNAs who experienced uncemented or hybrid THA, hybrid THA revision may provide a clinically significant improvement over uncemented THA revision with regard to implant survival, functional outcomes, and THA-related complications compared to uncemented THA revision.

## Background

Failure following proximal femoral nail antirotation (PFNA) for intertrochanteric hip fractures (IHFs) is rare and worrisome [1, 2]. Total hip arthroplasty (THA) tends to be a mainstay for managing PFNA failure [3, 4]. Patients with PFNA failure may be afflicted with a high risk of mortality, systemic or local complications, and reduced health-related quality of life. Revision to THA has been an acknowledged and multifaceted salvage procedure for a failed PFNA [5, 6]. With the burden of the secondary procedure of revision PFNA expected to increase at an incredible rate with the ageing population and expanding age range, revision to THA is becoming increasingly common [5]. However, the secondary procedure is frequently accompanied by significant loss of bone stock and increased stress in the acetabular and femoral sides, substantial bone wear, and damage to soft-tissue tensioning, making revision to the THA procedure challenging [4, 5]. Furthermore, THA revision commonly entails specific implants and relatively sophisticated surgical techniques, involving bone grafting and additional cerclage wire fixation. Thus, uncemented or hybrid THA (the femoral component was cemented) revision following healed IHF fixation may result in increased risks of THA-related complications compared with primary uncemented or hybrid THA revision [7, 8].

Advocates of hybrid THA revision perceive benefits in clinical outcomes when compared with uncemented THA revision [9]. A retrospective study [10] of 115 cemented stems demonstrated enhanced clinical outcomes with at least 17 years of follow-up, and survival to revision for any reason was 86.1% (95% confidence interval [CI], 79.8–92.4%). A previous study [11] of 72 patients who experienced hybrid THA revision following PFNA failure showed that hybrid THA revision had acceptable clinical outcomes, with a THA-related complication rate of 20.8%. Despite these promising results attributed to hybrid THA, compelling data on long-term implant survival remain lacking, as sufficient study subjects are needed to support this outcome [12, 13]. Additionally, there are still ongoing concerns that hybrid THA revision has more surgery-related complications than uncemented THA revision and tends to be associated with the increased risks of osteolysis-related loosening initiated by abrasion of the bone cement and instability secondary to poor soft tissues, which may frequently resort to revision to reverse this situation [14, 15].

Evidence-based consensus measures on the prevention of THA-related complications fail to be detailed for revision to uncemented or hybrid THAs [16, 17]. Presently, there is very little information available, as far as we know, on the incidence of THA-related complications, survivorship, or functional outcomes with THA conversion in the elderly population. Hence, we performed a multicentre retrospective cohort study to evaluate the clinical outcomes of uncemented or hybrid THAs following PFNA failure in elderly individuals.

## Methods

### Study population

Consecutive elderly patients with failed PFNAs undergoing an uncemented or hybrid THA (ICD-9 CM code 81.51) revision between January 2008 and December 2019 were retrospectively identified from two medical centres. The type of femoral fixation in conversion arthroplasty is primarily based on the patient’s age, bone conditions (osteoporosis, bone defects, complex anatomy), and the risk of revision. The confirmation of the type of and reason for the secondary procedure (conversion to THA) was executed by 2 high-volume surgeons (WY and XZ), and the secondary procedure was executed by 4 high-volume surgeons as per consensus guidelines. Analogous postrevision rehabilitation programs were executed in all patients. Key inclusion criteria included patients aged ≥ 65 years old; primary IHF (AO/OTA 31. A1-2) treated using PFNA, followed by unilateral uncemented or hybrid THA. The details of the implant components are shown in Table 1. Key exclusion criteria included unavailable patient characteristics (e.g., bone mineral density [BMD], body mass index [BMI], indication for revision PFNA and/or THA revision); IHFs (AO/OTA 31. A3); inability to ambulate independently before IHFs occurred; loss to follow-up; tuberculosis-related diseases (e.g., meningitis, pleurisy, arthritis, tenosynovitis, and sheath strain); metabolic bone diseases (e.g., rickets initiated by long-term use of anticonvulsants or aluminium hydroxide, vitamin D deficiency rickets, renal osteodystrophy, and renal tubular acidosis); cardiopulmonary insufficiency requiring medication or medical intervention (e.g., pneumoconiosis, chronic pulmonary heart disease, and coronary artery disease); cerebrovascular events within 6 months; active infectious diseases (e.g., hepatitis); malignant tumours; and schizophrenia.

**Table 1:** Manufacturer details of implants

	Stem	Cup	PFNA
Uncemented THA(*n* = 116)	Corail^a^(uncemented femoral components)	Continuum^b^(uncemented cup)	Synthes, Solothurn, Switzerland
Hybrid THA (*n* = 120)	Exeter^c^(cemented femoral components)	Continuum^b^(uncemented cup)	Synthes, Solothurn, Switzerland

*THA* Total Hip Arthroplasty, *PFNA* Proximal Femoral Nail Anti-rotation

^a^DePuy, ^b^ZimmerBiomet, Warsaw, IN, USA, ^c^Stryker

### Outcomes and variables

Data collection was conducted by the coauthors (WY and XZ). The primary outcome was implant survival after THA revision. Secondary outcomes included the functional outcomes assessed using the Harris Hip Score (HHS) and the incidence of key THA-related complications. The endpoints in Kaplan–Meier survivorship included prosthesis revision, prosthesis loosening, and periprosthetic fractures. The time since THA revision was considered the underlying timescale in the time-to-event analysis. Patients were followed up at 1, 6, 9, and 12 months after THA revision and yearly thereafter until revision THA or THA failure, death, or end of the study. Revision was defined as an exchange or removal of at least one component [7]. Indications for THA revision included instability (such as, nonunion, periprosthetic femoral fracture and aseptic necrosis of the femoral head), mechanical failure (such as, cutout), and both. The definition of radiographic loosening was in accordance with previous descriptions [18].

### Statistical analysis

Categorical variables (sex, side, mechanism of IHFs, fracture type, comorbidities, indication for THA revision, conversion interval, and key THA-related complications) were compared with the chi-square test; continuous variables (age, BMI, BMD, and follow-up time) were compared with Student’s t test or Mann–Whitney U test. Kaplan–Meier curves were constructed to assess the survival outcomes. The comparison of the survival curves was executed using a log-rank test. The estimation of hazard ratios (HRs) with 95% CIs was executed using Cox proportional hazards modelling. The threshold of the *p* value was 0.05. Data analyses were implemented with SAS 9.4 (Cary, NC).

## Results

**Fig. 1:**
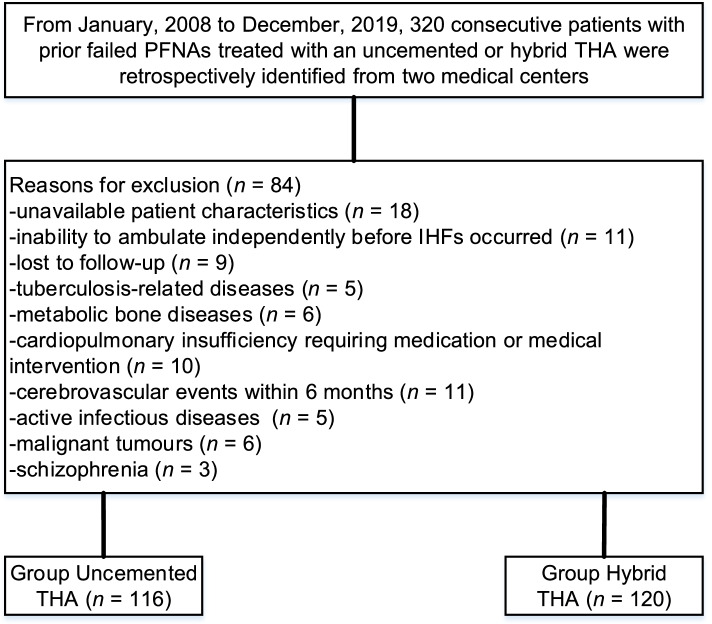
Flow diagram exhibiting methods for the identification of study population to evaluate the clinical outcomes of the conversion of failed PFNAs to uncemented or hybrid THAs in the elderly population

A total of 320 consecutive individuals with a prior failed PFNA treated with uncemented or hybrid THAs were retrospectively identified from three medical centres, 84 of whom were excluded, leaving 236 individuals (uncemented THA, *n* = 116; hybrid THA, *n* = 120) eligible for this study. Figure 1 demonstrates the methods for the identification of the study population. Patient characteristics at baseline between cohorts are shown in Table 2. The median age was 66.4 years (range, 65–71 years) in the uncemented THA group and 66.7 years (66–72 years) in the hybrid THA group. The hip condition prior to conversion was symptomatic for 56.9% and asymptomatic for 43.1% in the uncemented THA group versus symptomatic for 55.8% and asymptomatic for 44.2% in the hybrid THA group (*p* = 0.869). The femoral head size was 28 mm in 19.8%, 32 mm in 37.9%, and 36 mm in 42.2% of individuals in the uncemented THA group versus 28 mm in 21.7%, 32 mm in 34.1%, and 36 mm in 44.2% of individuals in the hybrid THA group (*p* = 0.945). The Charlson comorbidity index at surgery was low in 36.2%, medium in 49.1%, and high in 14.7% of individuals in the uncemented THA group versus low in 37.5%, medium in 50.0%, and high in 12.5% of individuals in the hybrid THA group (*p* = 0.717). Reasons for conversion were instability in 47.4%, mechanical failure in 38.8%, instability and mechanical failure in 13.8% of individuals in the uncemented THA group versus instability in 52.5%, mechanical failure in 35.0%, and instability and mechanical failure in 12.5% of individuals in the hybrid THA group (*p* = 0.459). The median follow-up was 120.2 months (102–127 months) in the uncemented THA group and 120.1 months (101–127 months) in the hybrid THA group.

**Table 2:** Patient characteristics at baseline between cohorts

Variable	Uncemented THA (*n* = 116)	Hybrid THA (*n* = 120)	*P*-value
Age (years)			
Median (range)	66.4(65–71)	66.7(65–72)	0.206
Sex, no. %			0.909
Female	55(47.4)	56(46.7)	
Male	61(52.6)	64(53.3)	
BMI (kg/m^2^)			
Median (range)	24.5(16.7–31.3)	24.2(16.1–32.8)	0.325
BMD	-3.61 ± 0.72	-3.64 ± 0.76	0.281
Side, no.%			0.443
Right	58(50.0)	54(45.0)	
Left	58(50.0)	66(55.0)	
Hip condition prior to conversion, no.%			0.869
Symptomatic	66(56.9)	67(55.8)	
asymptomatic	50(43.1)	53(44.2)	
Femoral head size (mm), no. %			0.945
28	23(19.8)	26(21.7)	
32	44(37.9)	41(34.1)	
36	49(42.2)	53(44.2)	
IHFs, AO/OTA, no. %			0.543
31A1	48(41.4)	45(37.5)	
31A2	68(58.6)	75(62.5)	
Charlson comorbidity index at surgery, no. %			0.717
Low	42(36.2)	45(37.5)	
Medium	57(49.1)	60(50.0)	
High	17(14.7)	15(12.5)	
Reasons for conversion, no. %			0.459
Instability	55(47.4)	63(52.5)	
Mechanical failure	45(38.8)	42(35.0)	
Both	16(13.8)	15(12.5)	
Conversion interval (years)			0.644
< 2	74(63.8)	80(66.7)	
≥ 2	42(36.2)	40(33.3)	
Follow-up (months)			
Median (range)	120.2(102–127)	120.1(101–127)	0.219

*THA* Total Hip Arthroplasty, *BMI* Body Mass Index, *BMD* Bone Mineral Density

### Primary outcome

**Fig. 2:**
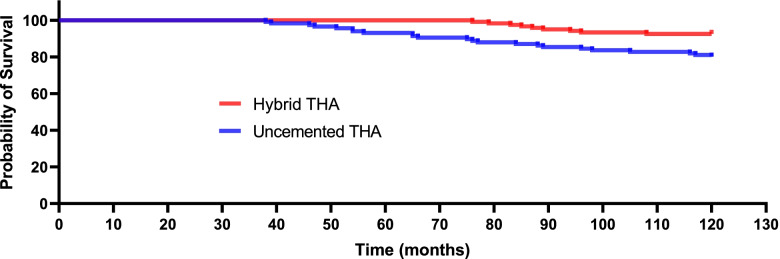
Kaplan–Meier survival curve for both cohorts with prosthesis revision for any reason as and an end point

At the final follow-up, 25 patient deaths occurred in the uncemented group, and 22 deaths occurred in the hybrid group(*p* = 0.537). With prosthesis revision for any reason as an outcome, the survival time in the uncemented THA group was markedly shorter than that in the hybrid THA group. Figure 2 shows the Kaplan–Meier survival analysis for both cohorts with prosthesis revision for any reason as an outcome. The most common indications for revision THA were aseptic loosening (femoral component) and periprosthetic fractures. The Kaplan–Meier estimate revealed that the 10-year cumulative survival rates were 0.801 (95% CI, 0.783–0.852) in the uncemented THA group versus 0.925 (95% CI, 0.861–0.964) in the hybrid THA group (HR 0.36 [95% CI 0.24–0.56], *p* = 0.004).

### Secondary outcomes

**Fig. 3:**
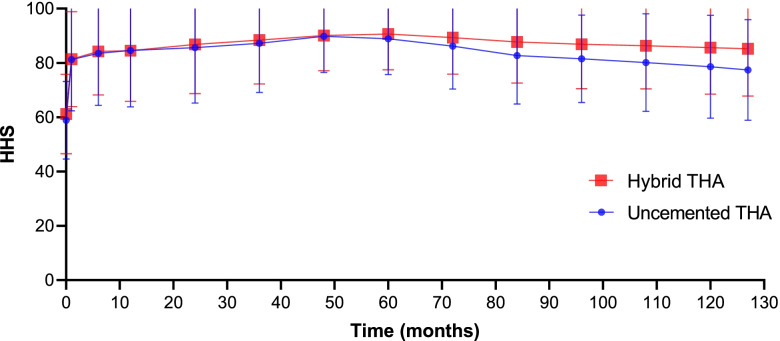
The change curve of the mean value of both cohorts of functional outcomes at each follow-up

**Fig. 4:**
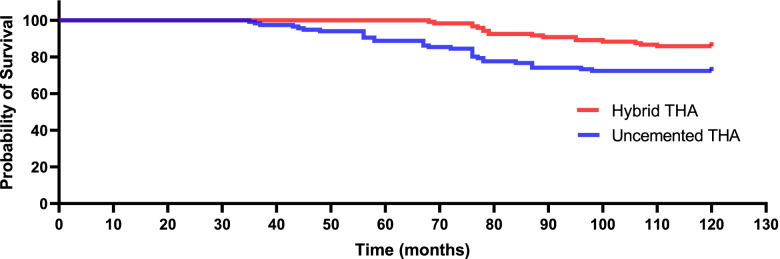
Kaplan–Meier survival curve for both cohorts with prosthesis loosening as an end point

**Fig. 5:**
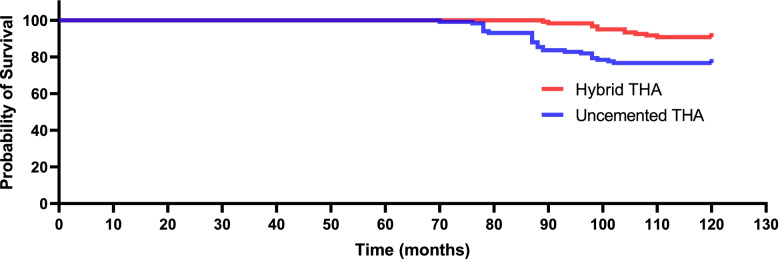
Kaplan–Meier survival curve for both cohorts with periprosthetic fractures as an end point

Table 3 shows the long-term, follow-up functional outcomes. Figure 3 characterises the variation trend of the HHS. From the 72nd month following conversion to THA to the last follow-up, functional outcomes differed considerably between cohorts (each *p* < 0.05). A significant difference was seen at the final follow-up (77.4 ± 13.1 in the uncemented THA group vs. 85.2 ± 12.3 in the hybrid THA group, *p* = 0.014). Furthermore, the trend of divergence gradually increased over time. Table 4 shows the long-term follow-up of key THA-related complications. Ninety-five key THA-related complications in 39 patients were seen in the uncemented THA group versus 44 in 21 patients in the hybrid THA group. The overall rates of key THA-related complications were 33.6% (39/116) in the uncemented THA group versus 17.5% (21/120) in the hybrid THA group (*p* = 0.004). Figures 4 and 5 show Kaplan–Meier survival curves for both cohorts with prosthesis loosening or periprosthetic fractures as end points, respectively. In the uncemented THA group, 23 (19.8%) patients underwent revision THA, 32 (27.5%) had aseptic loosening, and 27 (23.2%) were affected by a periprosthetic fracture. In the hybrid THA group, 9 (7.5%) patients experienced revision THA, 17 (14.1%) had aseptic loosening, and 11 (9.1%) were affected by a periprosthetic fracture. Eleven aseptic loosening (femoral side) and 6 periprosthetic fractures resulted in revision THA in the uncemented THA group; two aseptic loosening (femoral side) and 1 periprosthetic fracture resulted in revision THA in the hybrid THA group (73.9% [17/116] vs. 33.3% [3/120], *p* = 0.001).

**Table 3:** Long-term follow-up functional outcomes

Month(s) after revision	Uncemented THA (*n* = 116)	Hybrid THA (*n* = 120)	*P*-value
1	81.2 ± 13.3	81.4 ± 12.4	0.102
6	83.5 ± 13.5	84.2 ± 11.3	0.136
12	84.6 ± 14.7	84.5 ± 13.2	0.213
24	85.6 ± 14.4	86.8 ± 12.8	0.372
36	87.4 ± 13.0	88.4 ± 11.4	0.291
48	89.8 ± 9.4	90.1 ± 9.2	0.106
60	88.9 ± 9.3	90.6 ± 9.3	0.116
72	86.2 ± 11.2	89.3 ± 9.5	0.027*
84	82.7 ± 12.6	87.7 ± 10.7	0.015*
96	81.5 ± 11.4	86.9 ± 11.6	0.032*
108	80.1 ± 12.7	86.3 ± 11.2	0.012*
120	78.6 ± 13.4	85.6 ± 12.1	0.017*
Final follow-up	77.4 ± 13.1	85.2 ± 12.3	0.014*

*THA* Total Hip Arthroplasty

^*^Statistically significant values

**Table 4:** Long-term follow-up of key THA-related complications

Variable, No. %	Uncemented THA (*n* = 116)	Hybrid THA (*n* = 120)	*P*-value
Revision	23(19.8)	9(7.5)	0.006*
Aseptic loosening^a^	32(27.5)	17(14.1)	0.011*
Periprosthetic fractures	27(23.2)	11(9.1)	0.003*
Dislocation	5(4.3)	4(3.3)	0.695
Intolerable hip pain	7(6.0)	3(2.5)	0.178

*THA* Total Hip Arthroplasty

^a^occurred in femoral side

^*^Statistically significant values

## Discussion

The most important findings of this study were that with a 10-year follow-up, conversion to hybrid THA may have markedly improved 10-year survival, higher HHSs, and a lower incidence of key THA-related complications in elderly patients with a failed PFNA compared with conversion to uncemented THA. Nevertheless, the total amount of hybrid THAs was relatively small in the present study, despite more than 100 THAs. To reduce the risk of selection bias associated with retrospective design towards complex patients undergoing uncemented or hybrid THAs, we adjusted the baseline variables in the Cox proportional hazards model. Overall, survivorship tended to favour hybrid THA over uncemented THA (92.5% at 10 years vs. 80.1% at 10 years, *p* = 0.001) with regard to the risk of revision primarily due to aseptic loosening (femoral component) or periprosthetic fractures. Furthermore, patients who experienced uncemented THA may have a significantly higher incidence of key THA-related complications at the final follow-up (33.6% vs. 17.5%, *p* = 0.004).

Our findings are consistent with previous literature [5, 9] that showed the superiority of hybrid THA over uncemented THA in elderly patients. A register study [1] of 347,899 patients experiencing THA during 1995–2011 showed that the reduced risk of revision hybrid THA was detected during the initial 6 months, regardless of age, when compared with uncemented THA, primarily owing to aseptic loosening or periprosthetic fractures, and the survival of hybrid THA was higher than that of uncemented THA in individuals aged 65 years or older. Similarly, the results [19] based on the Nordic Arthroplasty Register Association data showed that the survival superiority of hybrid THA over uncemented THA is remarkable in patients aged 65 years or older. In individuals aged 64 years or younger, uncemented THA fails to show an advantage in revision rate, but they tend to be associated with a lower long-term risk of revision secondary to implant loosening compared with hybrid THA, which is consistent with our findings. Furthermore, the results [14] based on the Australian Orthopedic Association National Joint Replacement Registry showed that hybrid THA had significant advantages in terms of long-term survival and revision rates in all age groups compared with uncemented THA.

The evidence on which the choice to utilise an implant to convert a PFNA was based was under debate [20]. In accordance with published studies [6, 21], survival did differ considerably between uncemented and hybrid THAs. The performance of uncemented THA provides inferior long-term survivorship that tends to be attributed to component instability associated with bearing surface wear and periprosthetic fractures, which may result in early failure [14, 22, 23]. Minor fissures could occur during uncemented stem insertion, which could explain why patients are more prone to suffering a periprosthetic fracture even after slight stress [24, 25]. A recent collaborative register study [26] showed that the utilisation of an uncemented femoral component may lead to a lower rate of periprosthetic fractures, but this finding has failed to be verified by registration data, and thus far, there is, for all we know, no other evidence that the uncemented femoral component would protect individuals experiencing revision to an uncemented THA from periprosthetic fractures. Based on previous generally recognised recommendations [27], uncemented THA is suitable for revision PFNA by young patients. Previous studies [9, 14, 22] have revealed that uncemented THA has remarkable advantages regarding THA-related complications in young individuals.

For elderly patients receiving conversion to uncemented or hybrid THAs, the survival of THAs may differ over time [1, 5]. In the present study, implant survival differed considerably during the initial 72 months of follow-up. An increasing body of evidence [3, 7] indicates that the early differences in implant survival between uncemented and hybrid THAs may be attributed to progressive pain instigated by component loosening that is associated with frequent microfracture and remodelling. Component loosening may be the leading mode of uncemented THA failure, as the revisions for progressive pain were executed earlier than revisions for other reasons [1, 18]. Elevated stress associated with the uncemented femoral component in the proximal femur may exacerbate or restructure the local cancellous bone structure and predisposes to early failure of the uncemented femoral component as a result of femoral prosthesis descent, aseptic loosening, periprosthetic fractures, or persistent pain [1, 5, 10]. Pain fails to resolve with time or may become progressively worse, which may be associated with bearing surface wear [1, 4, 14, 22].

Several limitations should be acknowledged when interpreting the findings of the current retrospective study. First, the retrospective design limits the ability to establish a causal link between the implant and the results. The exclusion of patients who were lost to follow-up or died may lead to overestimation of the cumulative incidence of an event in the company of competing risks, as the survival analysis assumes that the risk of end point events is independent of the risk of loss to follow-up or death. The selection bias associated with study design is primarily challenging for elderly populations with high mortality rates and in studies related to longer follow-up. In addition, assessment bias is inevitable, as the study design is not a double-blind design. Thus, the paucity of a sufficiently powered comparison of outcomes may have reduced the reliability of the conclusions. Second, the results may be constrained by the small numbers of patients, unfitting control of potential variables (surgeon volume, patient’s physical activity, and patient compliance), and wide time span. Third, some factors associated with prosthesis survival failed to be involved in the present analysis, such as distinctions in femoral head diameter and prosthesis positioning. Thus, the generalisability of the results may be lacking.

## Conclusions

This retrospective study aims to provide viable descriptive evidence that for the elderly population, hybrid THA may be superior to uncemented THA in the treatment of PFNA failure. Hybrid THA may provide improved functional outcomes, decrease the incidence of key THA-related complications, and prolong prosthesis survival. Our findings may contribute to the resolution of the enduring debate over revision PFNAs. Nevertheless, evidence-based on prospective studies is indispensable to confirm our conclusions.

## Data Availability

The datasets generated during and analyzed during the current study are not publicly available due to the protection of patient privacy but are available from the corresponding author on reasonable request.

## Abbreviations

PFNA: Proximal femoral nail anti-rotation
THA: Total hip arthroplasty
HHS: Harris Hip Score
CI: Confidence interval
HR: Hazard ratio
IHFs: Intertrochanteric hip fractures
BMD: Bone mineral density
BMI: Body mass index
